# Photosensitive and Flexible Organic Field‐Effect Transistors Based on Interface Trapping Effect and Their Application in 2D Imaging Array

**DOI:** 10.1002/advs.201500435

**Published:** 2016-02-26

**Authors:** Yingli Chu, Xiaohan Wu, Jingjing Lu, Dapeng Liu, Juan Du, Guoqian Zhang, Jia Huang

**Affiliations:** ^1^ School of Materials Science and EngineeringTongji UniversityShanghai201804P. R. China

**Keywords:** flexible electronics, interface trapping effect, organic field‐effect transistors, photosensitive

## Abstract

**Flexible organic phototransistors** are fabricated using polylactide (PLA), a polar bio­material, as the dielectric material. The charge trapping effect induced by the polar groups of the PLA layer leads to a photosensitivity close to ≈10^4^. The excellent performance of this new device design is further demonstrated by incorporating the photo­transistors into a sensor array to successfully image a star pattern.

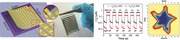

The charge trapping effect has been generally considered as a pitfall for organic electronics. However, we demonstrate that this effect can be subtly applied to enhance the photosensitivity of organic phototransistors (OPTs). The photosensitivity of traditional OPTs mainly arises from the organic semiconductor itself. Here we showed that it is possible to induce photosensitivity by engineering the organic semiconductor/dielectric interface of the device. Flexible OPTs were successfully fabricated using polylactide (PLA), a polar biomaterial, as the gate dielectric material. The polar groups of the PLA layer induce charge trapping effect at the organic semiconductor/dielectric interface, which results in reduced drain current when the device is in the dark. Under white light illumination, a significant increase in the drain current was observed, leading to a photosensitivity close to 10^4^. Even when ultralow illumination (≈0.02 mW cm^−2^) was used, the OPT based on this interface interaction still exhibited decent photosensitivity. The excellent performance of this new OPT design was further demonstrated by incorporating the devices into a 2D 10×10 array to successfully image a star pattern. The results indicate that the flexible PLA‐based OPTs with interface trapping effect could have promising applications, such as flexible and implantable photosensors, environmentally friendly electronics, and artificial skin.

Organic field‐effect transistors (OFETs) have attracted considerable research attention due to their potential application for low‐cost, flexible, and wearable electronics.^1^, ^2^, ^3^, ^4^, ^5^, ^6^, ^7^, ^8^, ^9^, ^10^, ^11^, ^12^, ^13^, ^14^ OPTs are an attractive category of OFETs, which can be used as photodetectors, light‐induced switches, light‐triggered amplifiers, and image sensors, amongst other applications.^15^, ^16^, ^17^, ^18^, ^19^, ^20^, ^21^, ^22^, ^23^ In most studies on OPTs, it was the organic semiconductor itself, which exhibited photosensitivity. Under illumination, photogenerated excitons in the OPT lead to increased charge density and enhanced drain current. In order to obtain good photosensitivity, high‐quality organic semiconductor materials, well‐controlled morphology, specially designed crystallinity or sometimes even complicated fabrication processes can be needed for the fabrication of the devices, such as the employment of single crystal organic semiconductors or fabricating micro/nanostructured devices.^23^, ^24^, ^25^, ^26^, ^27^, ^28^ Therefore, improving the photosensitivity of OPTs by other novel strategies is interesting and required. Moreover, there is a need for flexible photosensitive devices that possess good biocompatibility for various prospective biomedical applications.^29^, ^30^, ^31^, ^32^ For example, in order to study the optogenetic behavior of the animal nervous system, mechanically compliant, ultrathin multifunctional optoelectronic systems have been implanted into the brains of mice.^30^ However, the biocompatibility and flexibility of these kinds of devices, which were usually fabricated using polydimethylsiloxane (PDMS) and inorganic silicon‐based material, need to be further improved for a wide range of applications that require more biocompatibility, such as implantable photo sensors, bionic optical imaging equipment, and biodegradable electronic devices.

Here in this work, we utilized the organic semiconductor/dielectric interface interaction to enhance the photosensitivity of OPTs, and successfully fabricated flexible and biocompatible PLA‐based device by taking the advantage of the charge‐trapping effect. PLA is a biopolymer that exhibits excellent biocompatibility. It has been approved by the United States Food and Drug Administration (FDA) for human use.^33^, ^34^, ^35^, ^36^ We propose that an OPT device constructed using a PLA‐based gate dielectric would allow the polar groups of the PLA to interact with the organic semiconductor and induce interface charge trapping effect.^37^, ^38^, ^39^ The charge trapping effect has been generally considered a pitfall for organic electronics since it reduces charge mobilities and deteriorates device performances, but used here to enhance the photosensitivity of the fabricated OPT devices. When the photosensitivity is generated by the interface charge trapping effect more than the organic semiconductor itself, a wide range of organic semiconductors and fabrication methods can thus be adopted with fewer limitations than that of traditional photosensitive OFETs. The source–drain current of our PLA‐based OPT under illumination was enhanced by ≈10^4^ compared to the dark current, as well as the threshold voltage and charge carrier mobility changed significantly. We also demonstrated the application of our PLA‐based OPTs in a two‐dimensional array for image detection.

The PLA‐based OPTs were fabricated using a top‐contact geometry, as shown in **Figure** [qv: **1**]a. A silicon wafer was used as template substrate. The wafer surface was first treated using (tridecafluoro‐1, 1, 2, 2‐tetrahydrooctyl) trichlorosilane (FOTS) to create a self‐assembled monolayer that served as a release layer. Gold gate electrodes were subsequently evaporated onto the treated silicon substrate.^40^ Next, PLA and dinaphtho [2,3‐b:2′,3′‐f]‐thieno[3,2‐b] thiophene (DNTT) were deposited onto the treated silicon wafer as the dielectric and semiconductor materials, respectively.^41^ The chemical structures of PLA and DNTT are shown in Figure 1b. A solution of PLA in chloroform was deposited onto the substrate by dip coating to provide a 3 μm thick polar dielectric layer. The roughness of this PLA dielectric layer was found to be less than 1 nm (Figure S1a,b, Supporting Information). Organic semiconductor DNTT was then deposited onto the PLA layer by thermal evaporation. Source and drain electrodes were then deposited by thermally evaporating gold through a shadow mask. The configuration of the flexible PLA‐based OPT array is illustrated in Figure 1c. Using the FOTS as a release layer, the PLA‐based OPT array could be easily peeled off from the template silicon substrate. The template substrate could then be reused in the same fabrication process. The total thickness of the free standing device was less than 4 μm and thus it was quite light and highly flexible, as shown in Figure 1d.

**Figure 1:**
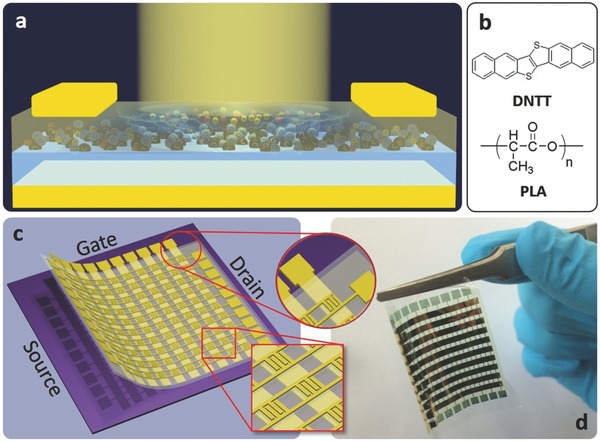
a) A schematic diagram of the OPT device structure. b) The chemical structure of the organic semiconductor (DNTT) and the dielectric (PLA) materials. c) A schematic presentation of the flexible OFET array configuration. d) An image of the flexible OFET array.

The photosensitivity of the PLA‐based OPT was investigated under dark and white light illuminated conditions (LED, Thorlabs MCWHL5‐C4). As shown in **Figure** [qv: **2**]a, the OPT exhibited good transistor *I*_d_‐*V*_d_ characteristics in the dark, displaying both a linear and saturation regime. At the same applied voltage, under illuminated conditions, the device exhibited a substantial increase in the drain current, *I*_d_, as shown in Figure 2b. Figure 2c shows the transfer characteristics of the OPT under various light intensities, with the drain voltage *V*_d_ fixed at −60 V. The saturation source–drain current *I*_d‐sat_ was significantly enhanced by illumination, even when the light was as weak as 0.5 mW cm^−2^. In fact, the OPT also exhibited photosensitivity to ultralow levels of light (0.02 mW cm^−2^), as shown in Figure S2e (Supporting Information), which suggests the excellent photosensitivity of the device. Figure 2d plots the device photosensitivity, which was defined as the normalized ratio between *I*_d‐sat_ under light and *I*_d‐sat_ in the dark (normalized *I*_light_/*I*_dark_), as a function of the gate voltage *V*_g_. Photosensitivity close to 10^4^ was achieved at low gate voltages. The amplification of the drain current under illumination was strongly dependent on the incident light intensity.

**Figure 2:**
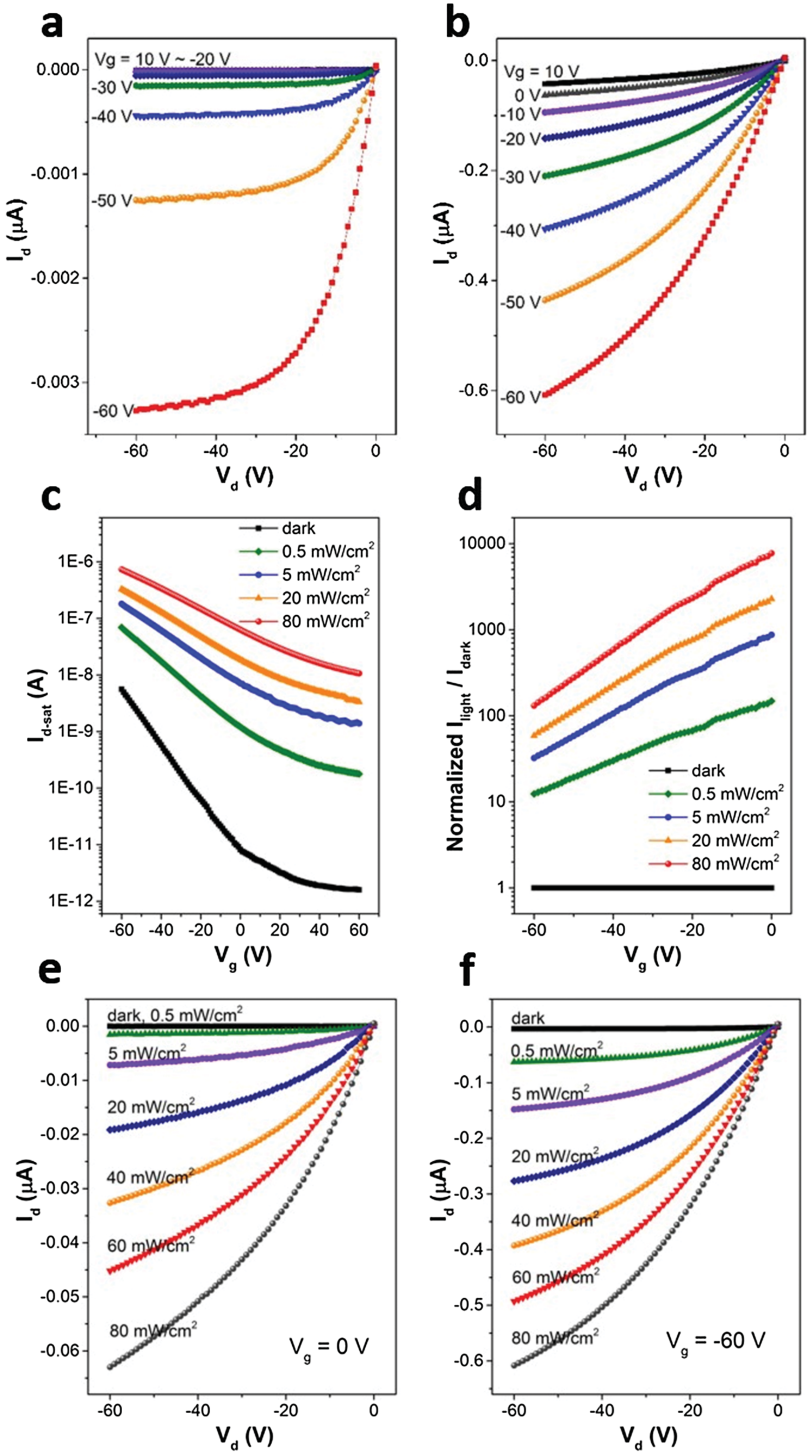
*I*_d_–*V*_d_ transistor characteristics of the OPT under fixed light intensity a) in dark and b) under 80 mW cm^−2^ light illumination. c) Transfer characteristics of the OPT under different light intensities. d) Normalized photosensitivity *I*_light_/*I*_dark_ as functions of *V*_g_ and light intensity. *I*_d_–*V*_d_ curves of the OPT at various light intensities with e) *V*_g_ fixed at 0 V, and f) *V*_g_ fixed at −60 V.

The OPT devices can also operate at a fixed *V*_g_ mode, with the output current modulated by light intensity. Figure 2e,f show the *I*_d_–*V*_d_ curves of the PLA‐based OPT under different intensities of incident light at a fixed *V*_g_ of 0 and −60 V, respectively (also see Figure S2a,b in the Supporting Information for results with *V*_g_ = −40 V and −20 V, respectively). For each applied gate voltage, output *I*_d_–*V*_d_ characteristics similar to that of typical p‐type OFETs were observed. The *I*_d_–*V*_d_ curves show both a linear and a saturation regime, but the light intensity, instead of the gate voltage, modulated the output *I*_d_. Figure S2c,d (Supporting Information) shows the log scale plot of Figure 2e,f. The OPT exhibited good performance even at low light intensity. At just 0.5 mW cm^−2^, the photosensitivity (*I*_light_/*I*_dark_) was shown to be over 100 and 10 at a fixed *V*_g_ of 0 V and −60 V, respectively. These combined results suggest that the output drain current can be controlled both by the gate voltage and the intensity of the incident light.^42^, ^43^ By changing the applied *V*_g_, the photosensitivity of the device can be controlled in a wide range, which provides additional modulation methods for practical applications.

It was assumed that the photosensitivity behavior of OPTs could be driven by capacitance change of dielectric, or photoinduced charge transfer, which is effectively affected by morphology and grain boundary within the active layer, or charge trapping process between semiconductor and dielectric interface.^44^, ^45^, ^46^ To further understand the photoresponse of our devices, capacitance variation of the PLA dielectric along with light intensity was measured and shown negligible response to light, as shown in Figure S3 (Supporting Information). In addition, the morphology of semiconductor film of the DNTT OFET with PLA dielectric layer and that of a DNTT OFET with octadecyltrichlorosilane (OTS)‐treated SiO_2_ dielectric layer were characterized by atomic force microscopy (AFM) as shown in Figure S1c,d (Supporting Information). The AFM images of DNTT deposited respectively on PLA dielectric and OTS‐treated SiO_2_ dielectric show that they have similar textures of small islands and similar size of grains. In contrast, the photosensitivity of the DNTT OFET with different dielectric exhibited obvious difference. **Figure** [qv: **3**]a shows the OFET with the OTS–SiO_2_ dielectric layer exhibits almost no photosensitivity, while the *I*_d‐sat_ of the PLA‐based OPT was enhanced by two orders of magnitudes between dark and illuminated environments at *V*_g_ = −60 V though they have very similar morphologies. At a lower gate bias, a more significant difference was observed for the two OPTs, in which case the *I*_d‐sat_ of the PLA‐based OPT was enhanced by more than 10^3^ times (*V*_g_ = −20 V, *V*_d_ = −60 V) under light, as shown in Figure 3b. Thus we believe that the enhanced photosensitivity of our PLA‐based OPT is induced by the interfacial charge trapping effect.

**Figure 3:**
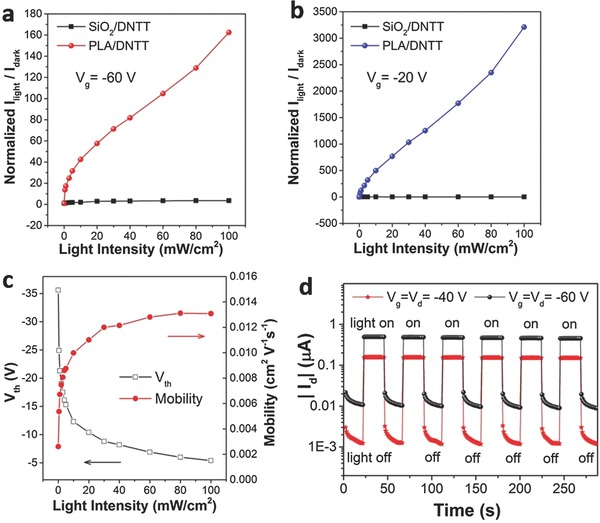
Comparison of normalized photosensitivity *I*_light_/*I*_dark_ between the PLA‐based OPT and the OFET with OTS‐modified SiO_2_ dielectric layer at gate voltages of a) −60 V and b) −20 V. c) Mobility and threshold voltage variations of the OPT as a function of light illumination intensity. d) Photo­response of the OPT with time at different applied voltages with 80 mW cm^−2^ light switching on and off.

More specifically, the enhanced photosensitivity behavior can be explained based on the multiple trap and release model.^39^, ^47^, ^48^, ^49^ Polar groups in the PLA film can induce high density charge traps at different energy levels at the organic semiconductor/dielectric interface, where the majority of charge carriers are concentrated.^14^ The deep traps can capture charge carriers and reduce the carrier density, while the shallow traps reduce the carrier transportation rate by temporarily trapping the carriers. This charge trapping effect results in an ultralow drain current when the device is in the dark, which is highly desired for OPTs. After the device exposed upon illumination, photoinduced excitons would be generated and supplement the conducting channel with charge carriers, acting as detrapping of the charge carriers on the interface. The higher the light intensity, the more the photoexcitons, which leads to a significant increase of *I*_d‐sat_ for the OPTs. In contrast, such polar groups are not present on the interface of the device fabricated with an OTS‐modified SiO_2_ dielectric layer, and thus the device shows almost no photosensitivity (Figure 3a,b). The effective charge carrier mobility and threshold voltage of the PLA‐based OPT was estimated from the device's transfer characteristics (see Figure S2f, Supporting Information), and plotted as a function of light intensity as shown in Figure 3c. Due to the interface charge trapping effect, the OPT showed low charge mobility and a high initial threshold voltage when the device was in the dark. Under weak light illumination (<10 mW cm^−2^), the mobility increases significantly and the threshold voltage shifts dramatically because of the photogenerated charge carriers. When the intensity of the incident light was further increased, the conducting channel tends to be saturated with charge carriers, thus there was less change of mobility and threshold voltage.

The above behavior was further studied by evaluating the device photosensitivity upon with different wavelength of incident light. Spectrum of UV–Vis (Figure S4a, Supporting Information) shows that DNTT possesses an absorption peak around 450 nm, which means in our device, photoexcitons can only be generated when the wavelength of the incident light is less than 450 nm. Correspondingly, a “cut‐off” change of the OPT *I*_on_/*I*_off_ ratio along with light wavelength was found also around 450 nm. In the range lower than that, the device presents high *I*_on_/*I*_off_ ratio, while in the range higher than 450 nm, the OPT shows very limited photosensitivity. This finding strongly supports our explanation as mentioned above.

In order to further substantiate the potential of the PLA‐based OPT in optoelectronic applications, we investigated our device's photoresponse upon on‐and‐off switching of light illumination at various *V*_g_ and *V*_d_ configurations. Figure 3d shows the reproducible and reversible drain current response to the switching of light. The response time of the device is about 50 ms (Figure S5, Supporting Information). In addition, the OPT devices also exhibited obvious photosensitivity when they were operated under low applied voltage (Figure S6a,b, Supporting Information). These results indicate that the PLA‐based OFET has reliably high photosensitivity and is therefore of interest from a practical application point of view.

Due to the high light sensitivity, the PLA‐based OPT is very suitable for optoelectronic applications such as photosensitive imaging. We demonstrated this application by incorporating the PLA‐based OPTs into a 10 × 10 array to sense an object. As shown in **Figure** [qv: **4**]a, a star pattern was imaged using the PLA‐based OPT array. The *I*_d‐sat_ of each OPT in the array was measured and normalized to the background *I*_d‐sat_ that was measured without the presence of the star pattern (light intensity = 4 mW cm^−2^). The OPTs in the array that were blocked by the star pattern displayed lower values of normalized *I*_d‐sat_, while the rest OFETs displayed the same values as the background *I*_d‐sat_. The output results were exhibited in a matrix form that displayed similar pattern as the star object (Figure 4b), which indicates the PLA‐based OPT array is reliable for photosensitive imaging. The blurred edges of the image are due to the partially blocked OPTs.

**Figure 4:**
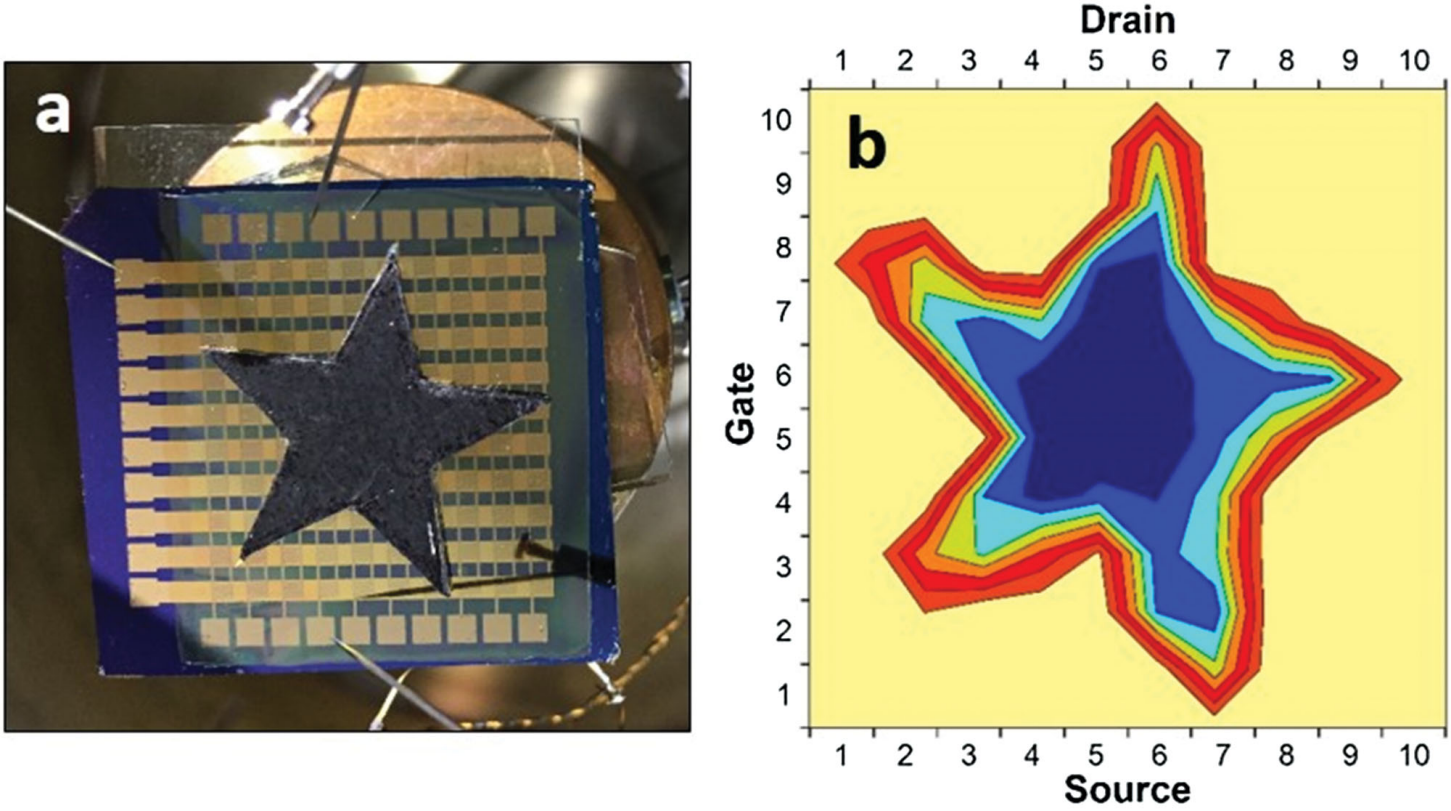
Pattern sensing behavior of the flexible OFET array: a) image of using the OFET array to sense a pentacle pattern. b) The output image of the pentacle by the OFET array.

In conclusion, by taking advantage of the interface charge trapping effect, flexible and biocompatible OPTs have been fabricated based on OFETs with a polar PLA layer acting as the dielectric material. The resulting OPTs showed good photosensitivity upon illumination as weak as 0.02 mW cm^−2^ and high photosensitivity (close to 10^4^) using high light intensity. In contrast with traditional OPTs, whose photosensitivity mainly arises from the organic semiconductor itself, the photosensitivity of our PLA‐based OPTs was mainly realized by engineering the organic semiconductor/dielectric interface of the device. The interface charge trapping effect has been generally considered a negative attribute in organic electronics, but here we subtly use the same effect to enhance the photosensitivity of OFETs. In order to demonstrate the reliable photosensitivity of our PLA‐based OPTs, the devices were incorporated into a 10 × 10 photosensing array and used to successfully image a star‐shaped object. Our results demonstrate a new strategy to develop high performance OPTs, which are applicable to many other organic electronics for sensing applications.

## Experimental Section

*Materials*: Polylactide was purchased from Natureworks Company and then recrystallized by ethyl acetate. FOTS were purchased from Sigma‐Aldrich. Organic semiconductor DNTT was synthesized according to literature reported previously, followed by vacuum sublimation purification.^41^

*OFET Devices Fabrication*: OFET devices were made using Si wafers as template substrate. FOTS chloroform solution (1, v/v%) was used to modified the substrates by spin coating at 3000 rpm for 20 s, followed by sonication cleaning in chloroform for 30 min. With the FOTS release layer, the transistor could be peeled off from silicon substrates easily after the fabrication process was completed. 80 nm gold gate electrodes were thermally evaporated onto the substrate after the deposition of the release layer. PLA dielectric films were made by dip coating 50 g L^−1^ PLA chloroform solution at a speed of 20 μm s^−1^ (Dip Coater, Shanghai SANYAN SYDC‐100H). The resulted films were dried at 60 °C in air overnight. The average thickness of PLA dielectric is 3 μm. After that, 60 nm DNTT film was deposited at the rate of 0.3 Å s^−1^ by vacuum thermal evaporation (*T*_substrate_ = 60 °C, *P* ≈ 5 × 10^−4^ Pa). 80 nm gold source–drain electrodes were thermally evaporated through a shadow mask to form top‐contact OFET devices. The channel length (*L*) and width (*W*) are 0.1 mm and 3.8 mm, respectively. OFETs with silica dielectric layer were fabricated for experiment control. Si wafers with 300 nm of thermally grown SiO_2_ layers were immersed in OTS toluene solution (1.2, v/v%) at room temperature for 3 h, followed by washed with toluene and ethanol. Subsequently, the substrate were annealed at 120 °C for 20 min and cleaned by sonication in toluene for 30 min. The OTS‐treated substrates were washed with ethanol and water and dried in flowing pure nitrogen. The deposition process of semiconductor and gold electrodes was the same as the fabrication of PLA‐based OFETs.

*Device Characterization*: The surface morphologies of PLA film and DNTT thin film are investigated by atomic force microscope (AFM, SEIKO SPA‐300HV) operated in tapping mode. PLA film and DNTT film (on quartz substrate) UV–vis spectra were recorded on a Cary‐60 UV–vis spectrophotometer (Agilent Technologies). For the characterizations of the devices under various illumination intensities, a LED (white light, Thorlabs MCWHL5‐C4) was employed as a light source and the illumination intensities were measured by an optical power meter (Thorlabs PM100D). Nine wavelengths of monochromatic light were obtained by the UV lamp (Uvata, Shanghai) and the LED irradiating through visible band‐pass filter kit (Thorlabs FKB‐VIS‐10), which were used to characterize the change of *I*_d‐sat_ along with wavelength of light. The light was illuminated from the top side of the devices. The measurement of PLA film capacitance was taken by a LRC bridge (TH2827C, Changzhou Tonghui Electronics Co., Ltd.). All electrical characterizations of OFETs were carried out under vacuum with a Keithley 4200‐SCS.

## Supporting information

As a service to our authors and readers, this journal provides supporting information supplied by the authors. Such materials are peer reviewed and may be re‐organized for online delivery, but are not copy‐edited or typeset. Technical support issues arising from supporting information (other than missing files) should be addressed to the authors.

Supplementary
