# Supplementary material for: Photosensitive and Flexible Organic Field‐Effect Transistors Based on Interface Trapping Effect and Their Application in 2D Imaging Array
Source: Adv Sci (Weinh). 2016 Feb 26;3(8):1500435. doi: 10.1002/advs.201500435 (PMC5069582; doi:10.1002/advs.201500435)
Supplement: Supplementary file 1 — Supplementary [file ADVS-3-0i-s001.pdf]

Copyright WILEY-VCH Verlag GmbH & Co. KGaA, 69469 Weinheim, Germany, 2016.

ADVANCED SCIENCE
Open Access

Supporting Information

for *Adv. Sci.*, DOI: 10.1002/advs. 201500435

Photosensitive and Flexible Organic Field-Effect Transistors Based on Interface Trapping Effect and Their Application in 2D Imaging Array

*Yingli Chu, Xiaohan Wu, Jingjing Lu, Dapeng Liu, Juan Du, Guoqian Zhang, and Jia Huang**

Copyright WILEY-VCH Verlag GmbH & Co. KGaA, 69469 Weinheim, Germany, 2013.

Supporting Information

Photosensitive and Flexible Organic Field-Effect Transistors Based on Interface Trapping Effect and Their Application in Two-dimensional Imaging Array

Yingli Chu[♣], *Xiaohan Wu*[♣], *Jingjing Lu, Dapeng Liu, Juan Du, Guoqian Zhang, and Jia Huang**

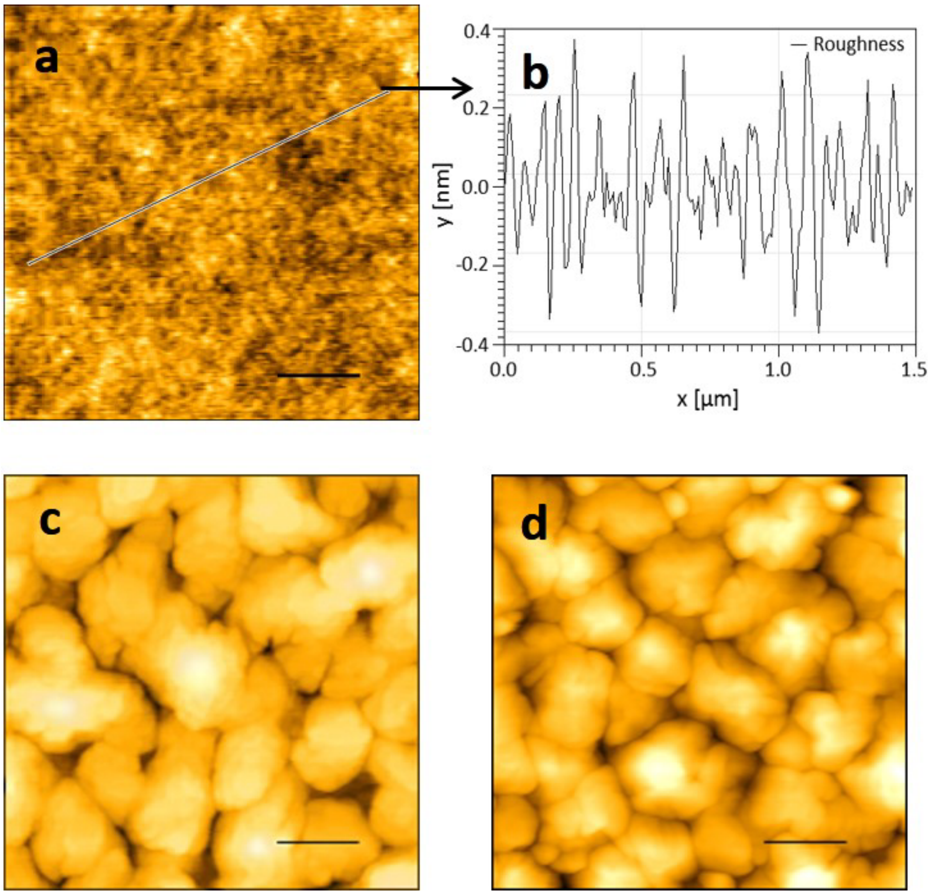

Figure S1. a) AFM height image of the PLA dielectric membrane. b) AFM line scan of the PLA membrane in (a). AFM height image of the DNTT layer deposited on c) OTS treated SiO_2 and d) PLA dielectric. Scar bars in (a), (c) and (d) are 300 nm.

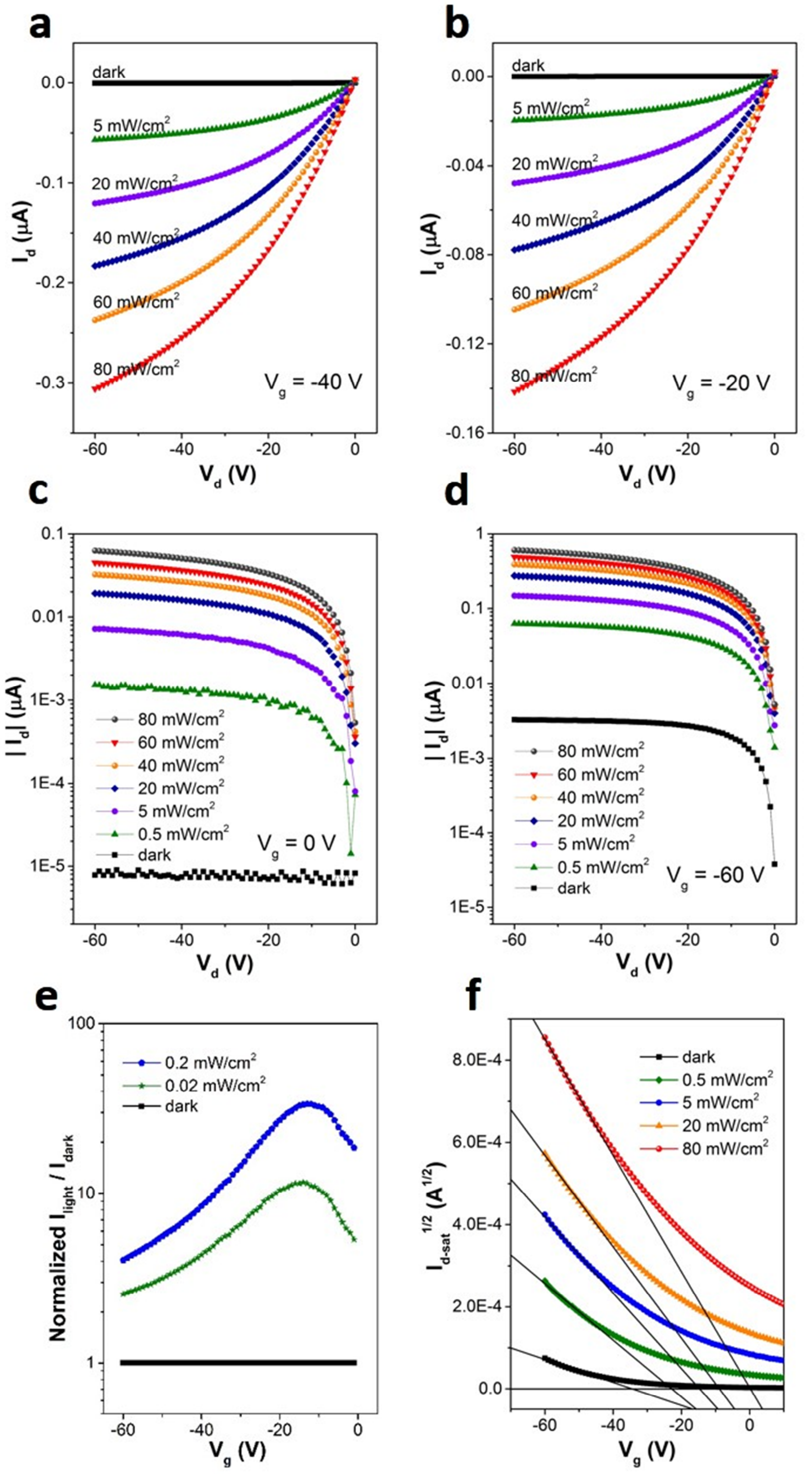

Figure S2. a) and b) Phototransistor behavior of the devices at different gate voltage. c) and d) Log scale plot of Figure 2e and 2f,respectively. e) Photocurrent under very low light density to dark current ratio. f) Plot of $I_{d\text{-}sat}^{1/2}$ vs V_g of the OPT under different light intensities.

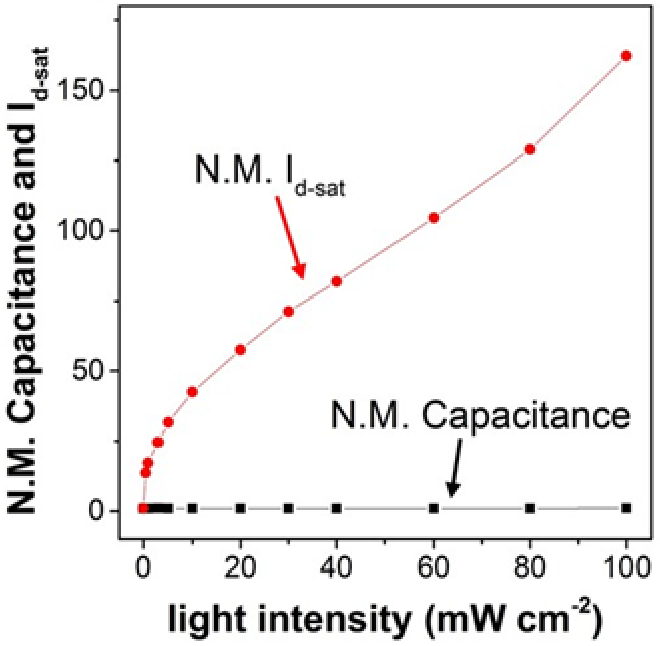

Figure S3. Variation of PLA dielectric capacitance as normalized to the value in darkness (N.M. capacitance) along with light intensity, compared with $I_{d\text{-}sat}$ of PLA based OPTs as normalized to the value in darkness (N.M. $I_{d\text{-}sat}$). The capacitances were measured at the frequency of 30 kHz.

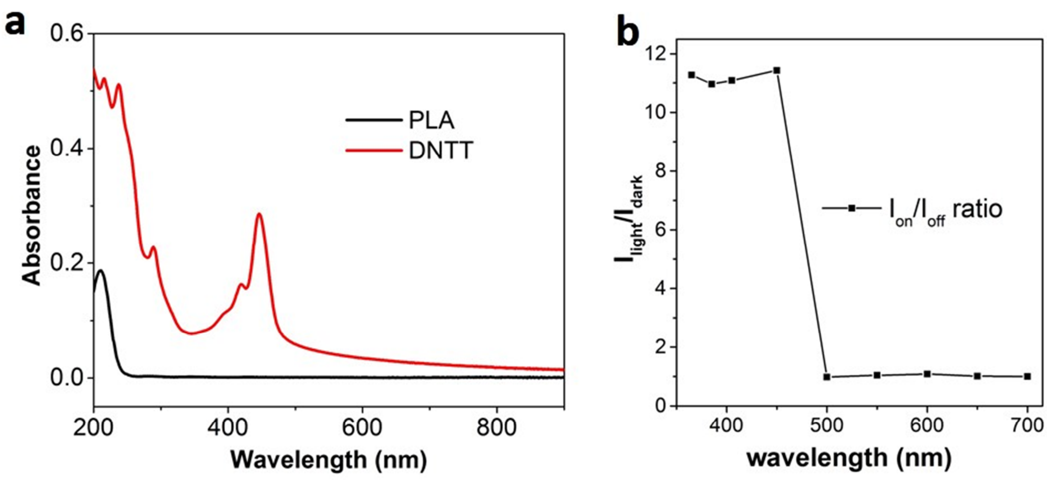

Figure S4. a) UV-vis absorption spectrum of the semiconductor DNTT film and PLA film, respectively. b) I_{light}/I_{dark} of the PLA-based OPT along with wavelength of light at 1.2 mW cm^{-2} (V_g = -60 V, V_d = -60 V).

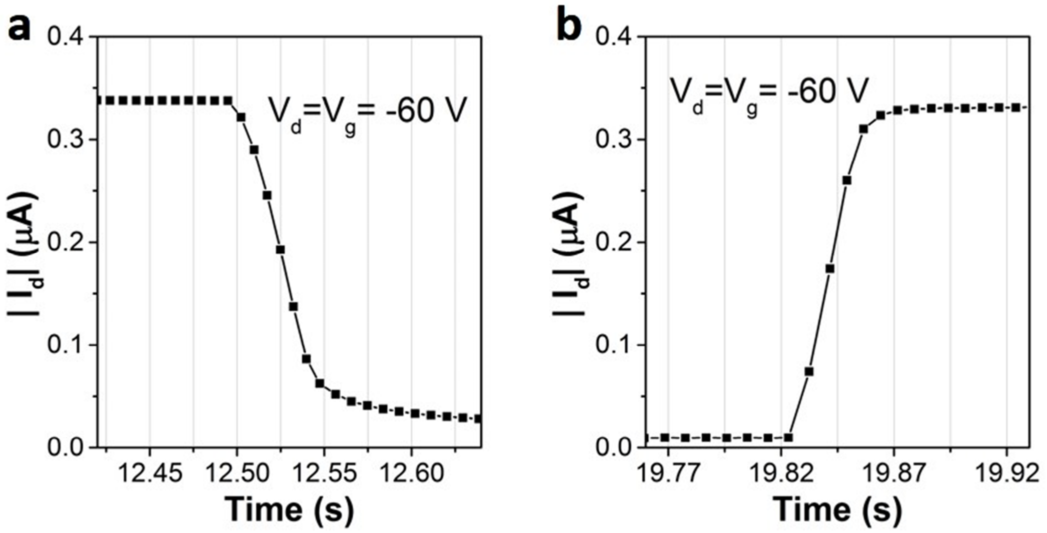

Figure S5. a) and b) Photoresponse time of OPTs as a function of time.

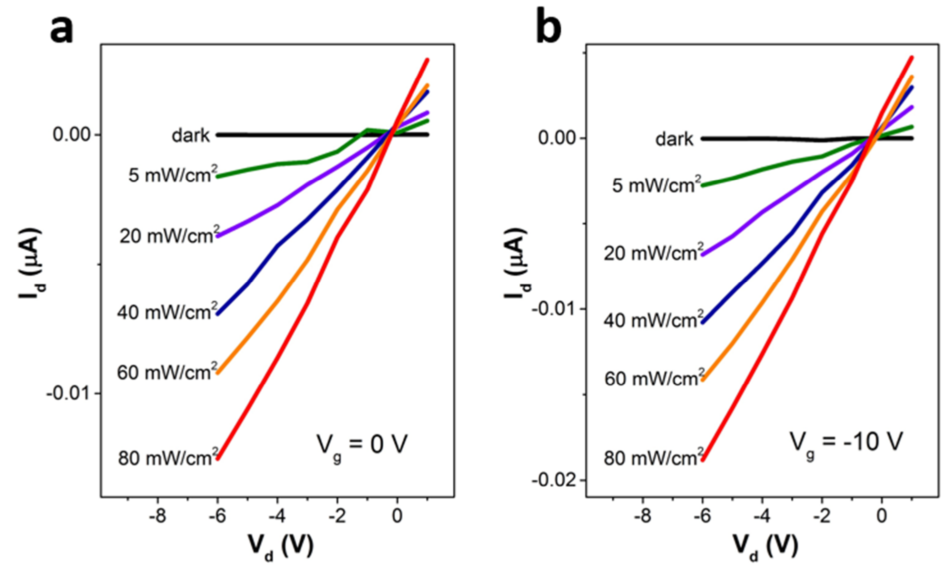

Figure S6. a) and b) Phototransistor behavior of the devices under low applied voltage .
